# Real-World Effectiveness, Tolerability, and Patient Satisfaction With a Hyaluronic Acid-Based Vaginal Moisturizer in Women With Vaginal Dryness: A Prospective, Multicenter, Observational Study

**DOI:** 10.7759/cureus.106828

**Published:** 2026-04-11

**Authors:** Manuel Sánchez-Prieto, Daniel María Lubián-López, María Eugenia Peña, José Subiris-González, Carmen Pingarrón Santofimia, Rafael Sánchez-Borrego

**Affiliations:** 1 Obstetrics and Gynecology, Instituto Universitario Dexeus, Barcelona, ESP; 2 Maternal and Child Health and Radiology, Faculty of Medicine, University of Cádiz, Cádiz, ESP; 3 Gynecology, Consulta de Ginecología, Vigo, ESP; 4 Gynecology, Consulta de Ginecología, Málaga, ESP; 5 Obstetrics and Gynecology, Universidad Europea de Madrid, Madrid, ESP; 6 Obstetrics and Gynecology, DIATROS, Clínica de Atención a la Mujer, Barcelona, ESP

**Keywords:** genitourinary syndrome of menopause, hyaluronic acid, moisturizer, real-world study, vaginal dryness

## Abstract

Objective: The objective of the study is to evaluate symptom improvement, safety, tolerability, and patient satisfaction associated with a hyaluronic acid-based internal vaginal moisturizer in women with symptomatic vaginal dryness under real-world conditions.

Methods: This prospective, multicenter, single-arm, open-label post-market clinical investigation was conducted in outpatient gynecology clinics in Spain between June and September 2022. Fifty-one women aged ≥18 years with symptomatic vaginal dryness were enrolled; 48 comprised the intention-to-treat population. Participants self-administered a CE-marked hyaluronic acid-based vaginal moisturizer three times weekly for six weeks. The primary endpoint was the change from baseline to week 6 in vaginal dryness severity measured using a non-validated 10-point visual analogue scale (VAS). Secondary endpoints included changes in irritation, itching, dyspareunia, vaginal hydration, vaginal pH, safety, and patient satisfaction. Within-subject changes were analyzed using paired comparisons.

Results: In participants with paired baseline and week 6 data (n = 47), mean vaginal dryness severity decreased from 7.34 ± 2.20 to 3.47 ± 2.00. The mean change was −3.87 points (95% confidence interval (CI) −4.62 to −3.12; t = −10.5; p < 0.001), corresponding to a large within-subject standardized effect size (Cohen’s dz = 1.54). Significant reductions were also observed for irritation (−2.11; n = 47), itching (−2.16; n = 45), and dyspareunia (−2.88; n = 42) (all p < 0.001). Vaginal hydration increased (+2.69; p < 0.001), and vaginal pH decreased modestly (−0.44; p = 0.001). Eight of 48 participants (16.7%) reported device-related adverse events, all mild to moderate; no serious adverse events occurred. Three participants discontinued due to adverse events.

Conclusions: In this prospective, real-world, single-arm clinical investigation, use of a hyaluronic acid-based vaginal moisturizer was associated with significant short-term reductions in vaginal dryness and related symptoms, with an acceptable safety profile. Given the absence of a comparator group, results should be interpreted cautiously. Findings should be considered hypothesis-generating.

## Introduction

Vaginal dryness is a prevalent and often underreported symptom affecting women across the reproductive lifespan, particularly during the menopausal transition and postmenopause [1,2]. As a core manifestation of genitourinary syndrome of menopause (GSM), vaginal dryness may be accompanied by irritation, pruritus, dyspareunia, and sexual dysfunction, with substantial impact on quality of life and intimate relationships [1,3]. Prevalence increases with advancing hypoestrogenism, reaching up to 50% several years after menopause, although symptoms may also occur in younger women exposed to hormonal suppression, cancer therapies, lactation, or certain medications [1,2,4-6].

Local estrogen therapy remains an effective treatment for GSM [7,8]. However, not all women are candidates for or willing to use hormonal therapy [9]. Non-hormonal vaginal moisturizers are therefore recommended as first-line therapy for mild-to-moderate symptoms and as an alternative when estrogen is contraindicated or declined [7,10]. These products aim to restore vaginal comfort through hydration, bioadhesion, and epithelial lubrication rather than hormonal modulation [10].

Hyaluronic acid-based formulations have gained attention due to their humectant properties, bioadhesive capacity, and potential to enhance mucosal elasticity [10-13]. Several studies suggest that hyaluronic acid may improve vulvovaginal symptoms; however, many are small, short-term, or methodologically heterogeneous, and variability in product composition (including pH, osmolarity, and excipients) may further contribute to differences in observed effectiveness and tolerability [10,11,14]. Many available data derive from short-term studies, heterogeneous populations, or controlled trial environments that may not reflect routine clinical practice.

Post-market clinical follow-up (PMCF) investigations conducted under real-world conditions are essential to characterize performance, safety, and patient satisfaction outside controlled trial settings. Such data are particularly relevant for medical devices intended for long-term or recurrent use in hypoestrogenic populations, where mucosal sensitivity may influence tolerability. The present prospective, multicenter clinical investigation was conducted to evaluate the effectiveness, safety, tolerability, and patient satisfaction associated with a CE-marked hyaluronic acid-based intravaginal moisturizer in women with symptomatic vaginal dryness under routine-use conditions.

## Materials and methods

Study design and setting

This was a prospective, multicenter, single-arm, open-label clinical investigation conducted under routine-use conditions to evaluate the effectiveness, tolerability, safety, and patient satisfaction associated with a CE-marked hyaluronic acid-based intravaginal moisturizer in women with symptomatic vaginal dryness. The study was designed as a PMCF investigation under Regulation (EU) 2017/745 on medical devices and conducted in accordance with International Organization for Standardization (ISO) 14155:2020.

Given the absence of a comparator arm, randomization, allocation concealment, and blinding procedures were not applicable. The investigation followed a pragmatic framework intended to reflect real-world gynecologic practice.

The study is reported in accordance with the CONSORT (Consolidated Standards of Reporting Trials) 2010 statement, adapted for non-randomized interventional studies, and the TIDieR (template for intervention description and replication) framework to ensure comprehensive reporting of intervention characteristics [15,16]. The investigation was conducted in outpatient gynecology clinics in Spain between June 13, 2022, and September 30, 2022, with follow-up completed at eight weeks.

Participants

Eligibility Criteria

Women aged ≥18 years presenting with symptomatic vaginal dryness were eligible. Symptoms could be associated with physiological or iatrogenic hypoestrogenic states, including menopause, climacteric transition, surgical menopause, lactation, hormonal contraception, cancer therapies, or other systemic treatments. Participants were required to report current vaginal dryness with or without associated symptoms, discontinue all vaginal treatments for at least seven days prior to baseline, refrain from using other intravaginal therapeutic products during the study period, and provide written informed consent.

Exclusion Criteria

Participants were excluded if they had active infectious vaginitis, known hypersensitivity to product components, recent initiation or modification of hormonal therapy, dermatologic or gynecologic conditions that could confound symptom assessment, participation in another clinical investigation, or anticipated non-adherence. Eligibility was confirmed during a structured clinical evaluation at baseline.

Investigational product

The evaluated formulation (Cumlaude Lab: Hidratante Interno®, Barcelona, Spain) is a CE-marked, non-hormonal vaginal moisturizer containing sodium hyaluronate, glycerin, and polyacrylic acid. The formulation has a pH range of 3.8-5.0 and is supplied in 5 mL single-dose applicators.

The product is designed to form a bioadhesive hydrating film over the vaginal epithelium, enhancing moisture retention and mucosal elasticity through humectant and film-forming mechanisms. The formulation contains no hormones. Full intervention details are reported according to TIDieR guidelines.

Intervention protocol

Participants self-administered one 5 mL intravaginal dose three times weekly, on alternate days, preferably at bedtime, for six consecutive weeks. Standardized written and verbal instructions were provided at baseline. Concomitant use of other vaginal moisturizers, lubricants, or medications was not permitted during the treatment period.

Study visits and follow-up

Participants were assessed at baseline (week 0), including eligibility confirmation, demographic and clinical data collection, symptom assessment, vaginal pH measurement, and product dispensing. At week 6, efficacy and safety outcomes were evaluated, including symptom scores, vaginal pH, hydration, and patient satisfaction. A telephone follow-up at week 8 was conducted for safety monitoring. In addition to scheduled visits, participants completed weekly symptom diaries throughout the six-week treatment period to capture the temporal evolution of vaginal dryness and associated symptoms.

Outcomes

Primary Endpoint

The primary endpoint was the change from baseline to week 6 in vaginal dryness severity measured using a 10-point visual analogue scale (VAS; 0 = none, 10 = worst imaginable).

Secondary Endpoints

Secondary endpoints included changes in irritation, itching, and dyspareunia severity (VAS), self-perceived vaginal hydration, vaginal pH, patient satisfaction, and safety and tolerability. VAS instruments were selected due to their simplicity, sensitivity to change, and frequent use in vulvovaginal symptom research.

Vaginal environment assessment

Vaginal pH was measured at baseline and week 6 using commercially available pH indicator strips applied to the lateral vaginal wall. Self-perceived vaginal hydration was assessed using a 0-10 VAS scale (0 = no hydration, 10 = maximum hydration).

Safety assessment

Adverse events (AEs) were defined as any untoward medical occurrence during product use, regardless of causal relationship. At each study contact, investigators recorded all AEs, classified severity (mild, moderate, or severe), assessed the relationship to the product (possible, probable), and documented duration and action taken.

Serious AEs were predefined according to the ISO 14155 criteria. Safety analyses included all participants who applied at least one dose (safety population).

Sample size considerations

A priori sample size estimation was performed for the primary endpoint (change in vaginal dryness severity from baseline to week 6). Assuming a standard deviation (SD) of 2.5 points for within-subject change in vaginal dryness based on previously published exploratory studies of non-hormonal vaginal moisturizers, a sample of 50 participants was estimated to provide 80% power to detect a 1-point mean change from baseline using a two-sided α of 0.05.

Because no universally accepted minimal clinically important difference (MCID) has been established for single-item VAS measures of vaginal dryness, the 1-point difference was selected pragmatically for sample size estimation. This threshold was considered conservative and intended to ensure adequate power to detect a statistically meaningful within-subject change rather than to confirm a predefined clinically validated MCID. To account for potential attrition, 51 participants were enrolled.

Statistical analysis

Analysis Populations

The modified intention-to-treat (mITT) population included participants with at least one post-baseline efficacy assessment. The per-protocol population included participants completing the week 6 efficacy assessment. The safety population included all participants receiving at least one application.

Weekly symptom assessments were prospectively collected to characterize the onset and trajectory of symptom changes during treatment. The primary prespecified analysis compared baseline and week 6 measurements. Intermediate weekly assessments were analyzed descriptively to illustrate temporal trends; no multiplicity adjustment was applied for these exploratory comparisons.

Statistical Methods

Continuous variables were summarized as mean ± SD, and categorical variables as frequencies and percentages. Within-subject changes from baseline to follow-up were assessed using paired t-tests for normally distributed variables or non-parametric alternatives where appropriate. Estimates of treatment effect are reported as mean change with 95% confidence intervals (CIs). Effect sizes were calculated using Cohen’s dz for paired samples.

All statistical tests were two-sided with α = 0.05. Analyses were performed using SAS software developed by SAS Institute (version 9.1.3, SAS Institute, Cary, NC, USA).

Missing Data

Missing data were not imputed. Analyses were conducted using available-case methodology for each comparison. Weekly diary analyses included only participants with both baseline and corresponding weekly measurements. No interim analyses were performed.

Ethics

The protocol was approved by the Grupo Hospitalario Quirónsalud-Catalunya Ethics Committee (May 24, 2022). The study was conducted in accordance with the Declaration of Helsinki, ISO 14155:2020, Regulation (EU) 2017/745 on medical devices, and applicable data protection regulations. All participants provided written informed consent prior to enrollment.

This PMCF clinical investigation was not prospectively registered in a public clinical trial registry because it was conducted as a pragmatic, non-randomized medical device performance study under routine-use conditions, and local regulatory/ethical requirements did not mandate public registration. The full protocol was finalized prior to enrollment.

## Results

Participant flow

Between June 13 and September 30, 2022, 51 women were enrolled across participating centers. All participants met eligibility criteria and initiated treatment.

Three participants were lost to follow-up after baseline and had no post-baseline efficacy data. The intention-to-treat (ITT) and safety populations, therefore, included 48/51 participants (94.1%). A total of 45/51 participants (88.2%) completed the week 6 visit. Three participants (3/51; 5.9%) discontinued treatment due to AEs.

**Figure 1:**
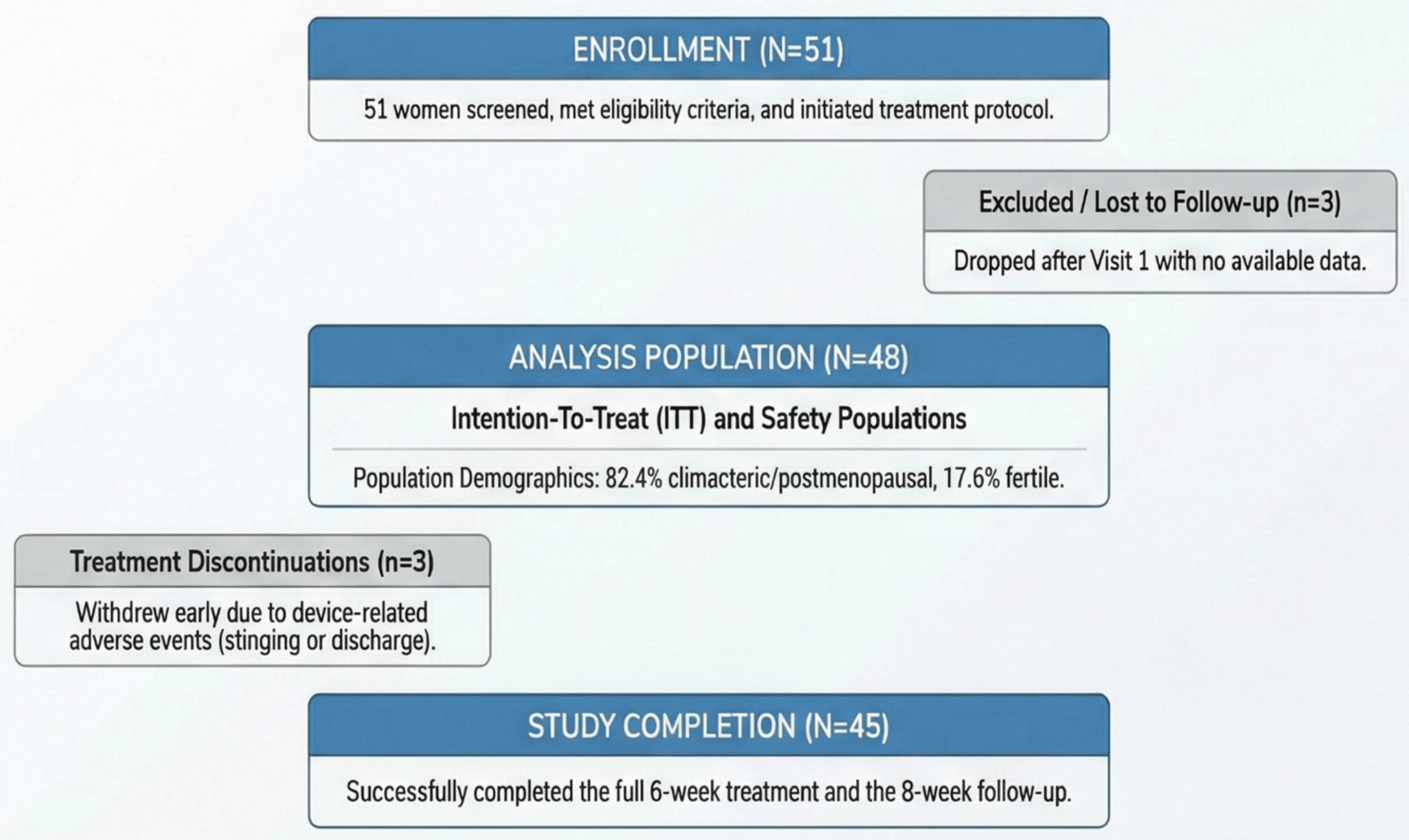
Participant flow diagram

Participant flow is shown in Figure 1.

Baseline characteristics

Baseline demographic and clinical characteristics are summarized in Table 1.

**Table 1:** Baseline demographic and clinical characteristics Data are presented as mean ± standard deviation (SD) unless otherwise indicated. Statistical significance was set at p < 0.05. *Categories for cause of vaginal dryness were not mutually exclusive. VAS: visual analogue scale (0 = no symptoms; 10 = worst imaginable symptoms)

Characteristic	Overall (N = 51)
Age (years)	
Mean ± SD	54.3 ± 10.1
Range	23-77
Hormonal status, n (%)	
Fertile	9 (17.6)
Climacteric/postmenopausal	42 (82.4)
Primary cause of vaginal dryness, n (%)*	
Menopause	36 (70.6)
Climacteric transition	2 (3.9)
Lactation/puerperium	2 (3.9)
Tamoxifen therapy	2 (3.9)
Hormonal contraception	3 (5.9)
Other systemic treatment	1 (2.0)
Hysterectomy	1 (2.0)
Other/unspecified	3 (5.9)
Baseline symptom severity (VAS 0-10), mean ± SD	
Vaginal dryness	7.22 ± 2.34
Irritation	4.63 ± 2.79
Itching (n = 49)	3.96 ± 2.68
Dyspareunia (n = 49)	6.80 ± 3.14
Vaginal hydration (n = 49)	3.80 ± 2.75
Vaginal pH	6.18 ± 1.01

The mean age was 54.3 ± 10.1 years (range 23-77). Most participants were climacteric or postmenopausal (42/51; 82.4%), while 9/51 (17.6%) were of reproductive age. Baseline vaginal dryness severity was high (mean VAS 7.22 ± 2.34), consistent with a symptomatic population.

Primary endpoint

In the ITT population with paired baseline and week 6 data (n = 47), mean vaginal dryness severity decreased from 7.34 ± 2.20 at baseline to 3.47 ± 2.00 at week 6. The mean within-subject change was −3.87 ± 2.52 points (95% CI −4.62 to −3.12; t = −10.5; p < 0.001), indicating a statistically significant reduction in symptom severity. The standardized effect size (Cohen’s dz) was 1.54 (95% CI 1.12 to 1.96), consistent with a large effect magnitude.

These results are summarized in Table 2.

**Table 2:** Change in vaginal dryness severity from baseline to week 6 (primary outcome) Data are presented as mean ± standard deviation (SD) or n (%). Statistical significance was set at p < 0.05. Within-subject comparisons were performed using paired t-tests. Effect size is expressed as Cohen’s dz for paired samples. Confidence intervals (CIs) for effect size were calculated using large-sample approximation. Analyses were conducted in the intention-to-treat population using available paired data. VAS: visual analogue scale (0 = no symptoms; 10 = worst imaginable symptoms)

Outcome	n	Baseline mean ± SD	Week 6 mean ± SD	Mean change (week 6–baseline) ± SD	95% CI of mean change	t-value	p-value	Effect size (Cohen’s dz)	95% CI of effect size
Vaginal dryness (VAS 0-10)	47	7.34 ± 2.20	3.47 ± 2.00	−3.87 ± 2.52	−4.62 to −3.12	−10.52	<0.001	1.54	1.12 to 1.96

**Figure 2:**
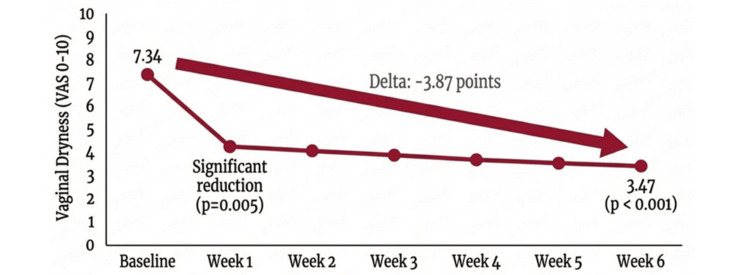
Weekly evolution of vaginal dryness severity during the 6-week treatment period Mean visual analogue scale (VAS) scores for vaginal dryness (0 = no symptoms; 10 = worst imaginable symptoms) from baseline through week 6. Error bars represent the standard error of the mean. Data are presented as mean ± standard error. Statistical significance was set at p < 0.05. Analyses include participants with paired baseline and weekly measurements in the intention-to-treat population.

The proportion of participants with moderate-to-severe dryness (VAS ≥ 7) decreased markedly by week 6, suggesting a potential clinically relevant shift toward lower symptom severity. The temporal evolution of vaginal dryness is shown in Figure 2. Exploratory descriptive analyses of weekly assessments suggested early symptom reduction from week 1 onward (p = 0.005, unadjusted for multiplicity); however, these comparisons were not prespecified as primary or secondary endpoints and should be interpreted with caution.

Secondary endpoints

**Figure 3:**
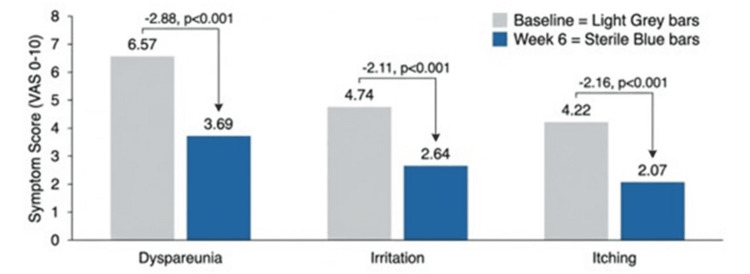
Mean change in secondary symptom severity from baseline to week 6 Mean within-subject change (±95% confidence interval) in irritation, itching, and dyspareunia severity measured using a 10-point visual analogue scale (VAS; 0 = none; 10 = worst imaginable). Negative values indicate symptom reduction. Data are presented as mean ± standard error. Statistical significance was set at p < 0.05. Analyses were conducted using paired data in the intention-to-treat population.

Secondary outcomes are summarized in Table 3 and illustrated in Figure 3.

**Table 3:** Changes in secondary outcomes from baseline to week 6 Data are presented as mean ± standard deviation (SD). Statistical significance was set at p < 0.05. Within-subject comparisons were performed using paired t-tests. VAS: visual analogue scale (0 = no symptoms; 10 = worst imaginable symptoms); CI: confidence interval

Outcome	n	Baseline mean ± SD	Week 6 mean ± SD	Mean change ± SD	95% CI of mean change	t-value	p-value	Effect size (Cohen’s dz)
Dyspareunia (VAS 0–10)	42	6.57 ± 3.10	3.69 ± 2.29	−2.88 ± 2.84	−3.77 to −1.99	−6.57	<0.001	1.01
Irritation (VAS 0–10)	47	4.74 ± 2.80	2.64 ± 1.86	−2.11 ± 2.59	−2.87 to −1.35	−5.58	<0.001	0.81
Itching (VAS 0–10)	45	4.22 ± 2.64	2.07 ± 1.74	−2.16 ± 2.77	−2.98 to −1.34	−5.23	<0.001	0.78
Vaginal hydration (VAS 0–10)	45	3.80 ± 2.64	6.49 ± 2.33	+2.69 ± 3.32	+1.70 to +3.68	5.45	<0.001	0.81
Vaginal pH	45	6.15 ± 1.01	5.71 ± 1.08	−0.44 ± 0.83	−0.69 to −0.19	−3.54	0.001	0.53

Ancillary (exploratory) analyses

Exploratory subgroup analyses suggested a greater reduction in vaginal pH among reproductive-aged women (−0.74) compared with climacteric/postmenopausal women (−0.36). No formal statistical comparison between subgroups was performed.

Patient satisfaction

At week 6, 43 participants provided satisfaction data. The mean satisfaction score was 7.02 ± 2.18. A total of 23/43 participants (53%) reported satisfaction scores ≥8/10, and 3/43 (7%) reported maximum satisfaction (10/10).

Safety and tolerability

Safety analyses included all participants who received at least one application (n = 48). A total of 8/48 participants (16.7%) reported at least one AE during the study period. All events were assessed as possibly or probably related to the investigational product. No serious AEs or deaths were reported.

Most AEs were mild to moderate and included transient vulvovaginal pruritus, stinging, or irritation following application. Additional events included excessive vaginal discharge and one case of non-specific vaginitis requiring treatment. One participant reported transient, strongly smelling urine that resolved spontaneously.

Three participants (3/48; 6.3%) discontinued treatment due to tolerability issues. All AEs resolved without sequelae. All reported events occurred in climacteric or postmenopausal participants.

Missing data

Missing data were not imputed. Denominators varied across outcomes due to loss to follow-up (n = 3), missing diary entries, and incomplete paired measurements at week 6. Analyses were conducted using available paired data for each endpoint.

## Discussion

In this prospective, multicenter, real-world clinical investigation, use of a hyaluronic acid-based intravaginal moisturizer was associated with a within-subject statistically significant reduction in vaginal dryness over six weeks, with a large standardized within-subject effect size. Symptom improvement was detectable as early as week 1 and progressed steadily throughout the treatment period.

The magnitude of dryness reduction (−3.87 points) falls within the range reported in prior studies evaluating non-hormonal vaginal moisturizers [7,10,11], where mean improvements of approximately 2-4 points on 10-point scales have been observed over short-term treatment intervals. Although no validated MCID has been established for single-item VAS measures of vaginal dryness, the observed effect size and distributional shift toward lower severity categories suggest a clinically meaningful change. However, interpretation must remain cautious: the uncontrolled design precludes attribution of improvement to the investigational product specifically, as placebo response, regression to the mean, and natural symptom variability may have contributed to the observed outcomes. Randomized controlled trials with an appropriate comparator are needed to confirm these findings.

Improvements were also observed across secondary symptom domains, including irritation, itching, and dyspareunia, all showing large standardized effect sizes. These findings are consistent with the multidimensional clinical presentation of vaginal dryness and align with previous studies evaluating hyaluronic acid-based formulations [10,11]. While randomized comparisons between hyaluronic acid-based moisturizers and low-dose vaginal estrogen have shown comparable short-term symptom relief in selected populations, the present study does not permit comparative inference [7].

The early onset of symptom improvement supports a mechanism of action primarily related to bioadhesive and humectant properties, leading to enhanced mucosal hydration rather than structural epithelial remodeling, which typically requires longer exposure to hormonal therapy. The modest reduction in vaginal pH (−0.44) may suggest partial normalization of the vaginal microenvironment; however, the clinical relevance of this finding remains uncertain in the absence of microbiological, cytological, or symptom-correlated data, and mechanistic conclusions cannot be drawn from pH measurement alone.

The investigational product demonstrated an acceptable short-term safety and tolerability profile, with no serious AEs and a low discontinuation rate. All reported AEs occurred in climacteric or postmenopausal women, which may reflect increased mucosal fragility in hypoestrogenic states [1]. However, the study was not powered to assess subgroup differences in safety.

Several strengths merit consideration. The prospective multicenter design, predefined endpoints, a priori sample size estimation, transparent reporting of analysis populations, and structured safety monitoring in accordance with ISO 14155 enhance internal validity. The pragmatic framework, including broad eligibility criteria, reflects routine gynecologic practice and supports external generalizability.

Limitations

Several limitations of this investigation must be acknowledged. First, the absence of a comparator arm precludes causal inference and prevents quantification of placebo response, expectation effects, and regression to the mean, all of which are known to contribute substantially to symptom improvement in uncontrolled studies of vulvovaginal symptoms. Second, the primary outcome was assessed using a single-item VAS, which lacks formal psychometric validation as a standalone measure of vaginal dryness severity; the absence of validated multidimensional instruments such as the DIVA questionnaire or the Most Bothersome Symptom approach limits construct validity and cross-study comparability. Third, follow-up was limited to six weeks, preventing assessment of the durability of response or long-term safety. Fourth, missing data were not imputed; the primary analysis was based on available paired observations, and although the missing data rate was low (2.1%), a missing-at-random assumption cannot be formally verified. Fifth, the etiologically heterogeneous population, while reflective of real-world practice, may limit subgroup interpretability given the small and unbalanced group sizes. Sixth, this investigation was not prospectively registered in a public trial registry prior to enrollment, which limits transparency and independent verifiability of pre-specified endpoints. Finally, as a manufacturer-sponsored PMCF study, potential influence on study conduct or reporting cannot be entirely excluded despite adherence to predefined methodological procedures and investigator-led analysis.

Within the constraints of a non-randomized design, these results suggest that a hyaluronic acid-based vaginal moisturizer may provide meaningful short-term symptomatic relief in women with vaginal dryness, including those in hypoestrogenic conditions. However, these findings do not establish comparative efficacy versus placebo, alternative non-hormonal therapies, or local estrogen treatment. Randomized controlled trials with longer follow-up and validated outcome measures are required to confirm effectiveness, assess durability, and define the role of this intervention within the therapeutic landscape of GSM.

## Conclusions

In this prospective, multicenter, single-arm clinical investigation conducted under routine-use conditions, use of a hyaluronic acid-based intravaginal moisturizer was associated with statistically significant reductions in vaginal dryness and related symptoms over a six-week treatment period. Improvements were observed early and were accompanied by increased perceived vaginal hydration and a modest reduction in vaginal pH. The product demonstrated an acceptable short-term safety and tolerability profile, with no serious AEs and a low discontinuation rate.

Given the non-randomized design, absence of a comparator group, and potential contribution of placebo response and regression to the mean, these findings should be interpreted with caution and cannot establish causal effectiveness. Randomized controlled trials with longer follow-up and validated multidimensional outcome measures are warranted to confirm the magnitude and durability of benefit and to define the comparative role of this intervention in the management of vaginal dryness and GSM. These findings should be considered hypothesis-generating.
